# Improvement in Inhaler Techniques After Training and Counseling in Patients With Chronic Obstructive Pulmonary Disease or Asthma

**DOI:** 10.7759/cureus.62255

**Published:** 2024-06-12

**Authors:** Muhammad Asad Abbas, Owais Tariq, Saad Bin Zafar, Muhammad Irfan Jamil, Khizra Hamid, Aqsa Iqbal, Adeel Ahmed, Iqra Naeem

**Affiliations:** 1 General Medicine, Pak Emirates Military Hospital, Rawalpindi, PAK; 2 Gastroenterology, Lahore General Hospital, Lahore, PAK; 3 Medicine, Milvik Bima Pakistan, Lahore, PAK; 4 Internal Medicine, King Edward Medical University, Lahore, PAK; 5 Nephrology, Lahore General Hospital, Lahore, PAK; 6 Internal Medicine, Evercare Hospital Lahore, Lahore, PAK; 7 Pulmonology, Lahore General Hospital, Lahore, PAK; 8 Medicine, Lahore General Hospital, Lahore, PAK; 9 Psychiatry and Behavioral Sciences, Lahore General Hospital, Lahore, PAK

**Keywords:** metered-dose inhaler (mdi), copd assessment test, dry powder inhalers, asthma, chronic obstructive pulmonary disease

## Abstract

Background

Chronic respiratory diseases such as chronic obstructive pulmonary disease (COPD) and asthma significantly impair quality of life and impose a substantial burden on healthcare systems. Proper inhalation technique is important for effective management of these diseases, yet remains poorly performed by many patients. This study evaluated the impact of structured counseling and training sessions on inhaler use among patients with COPD and asthma, aiming to enhance technique correctness and disease control.

Methodology

This cross-sectional study analyzed 150 patients with asthma and COPD who fulfilled the inclusion criteria for inhalation techniques. Patients were counseled regarding the proper seven-step inhalation technique for each inhaler type [metered-dose inhaler (MDI), MDI with spacer, and dry powder inhaler (DPI)] through practical demonstration at baseline visits. Correct use of inhalers was assessed by a predefined checklist for each inhaler device at the baseline visit and after three months. The correctness of the inhalation technique was evaluated by scoring each of the seven steps. The disease control assessment was done using the COPD assessment test (CAT) and asthma control test (ACT) at the baseline visit and after three months.

Results

In this study of 150 patients, there were 97 (64.7%) males and 53 (35.3%) females. In total, 67 (44.7%) were diagnosed with asthma and 83 (55.3%) with COPD. The mean age was 45.33 ± 12.62 years. Post-counseling improvements in inhaler technique were marked, with MDI users enhancing their technique score from an average of 4.4 to 6.1, MDI with spacer from 4.56 to 6.26, and DPI from 4.92 to 6.24 (p < 0.001 for all). Disease control also showed significant gains; CAT scores decreased for MDI users from 23.4 to 20.5, MDI with spacer from 23.92 to 20.96, and DPI from 24.89 to 21.96. Concurrently, ACT scores increased for MDI users from 16.4 to 18.0 (p = 0.002), MDI with spacer from 17.29 to 19.04, and DPI from 16.42 to 18.37 (p < 0.001 for both), reflecting substantive advances in managing COPD and asthma symptoms. Furthermore, patients with primary education exhibited a significant boost in technique mastery post-counseling (p < 0.001), underscoring the potential of well-crafted counseling to transcend educational barriers in promoting effective inhaler use.

Conclusions

Post-counseling, inhaler technique improved significantly across all types, with MDI with spacer users demonstrating the most progress. Technique scores increased notably (p < 0.001), and disease control scores for COPD and asthma, measured by CAT and ACT, also showed significant improvements (p < 0.001). Remarkably, primary education level participants exhibited substantial technique gains post-intervention, emphasizing the effectiveness of counseling irrespective of initial educational status in enhancing inhaler use and disease management.

## Introduction

Chronic obstructive pulmonary disease (COPD) and asthma are common chronic respiratory diseases that markedly affect public health [1]. COPD is defined as a heterogeneous lung disease characterized by chronic respiratory symptoms of dyspnea, cough, sputum production, and exacerbations due to abnormalities of the airways (bronchitis and bronchiolitis) and/or alveoli (emphysema) that causes persistent, often progressive, airflow obstruction. It reduces patients’ quality of life (QoL) and is a leading cause of morbidity and mortality worldwide [2]. Asthma is a chronic inflammatory disease of the lungs that affects the airways causing recurring episodes of wheezing, coughing, chest tightness, and breathlessness. These symptoms are associated with variable airflow obstruction and bronchial hyper-responsiveness [3].

Globally, around 339 million people suffer from asthma, and 1,000 people die daily due to this condition [4]. Approximately 251 million people are affected by COPD worldwide, which leads to 90% mortality in low- or middle-income countries [5]. In Pakistan, 15 million children and 7.5 million adults suffer from asthma, and 2.1% of the population is affected by COPD. These respiratory problems account for 25% of patients attending primary healthcare facilities in Pakistan [6]. By 2030, COPD and related conditions are expected to cause 4.5 million deaths worldwide. Smoking is a major risk factor for respiratory diseases [4].

Inhalation therapy is vital in treating asthma and COPD. It delivers medication directly into the airways, resulting in high local concentrations and fewer side effects. Inhalers such as metered-dose inhalers (MDIs), metered-dose inhalers plus spacer (MDIs + spacer), and dry powder inhalers (DPIs) are used for this therapy. Proper inhaler use is important to control COPD and asthma, deliver effective medication, improve disease control, and reduce hospitalizations [7].

Despite the availability of various medications, some studies suggest that a large number of COPD patients do not use their inhaler devices correctly. Errors in inhaler use may affect the efficacy of the drug delivery, thereby leading to the suboptimal control of COPD that causes multiple episodes of acute exacerbation which is associated with many morbidities and mortalities [8]. Inappropriate inhaler use can result in rising healthcare costs, medicine waste, and less effective drug delivery to the lungs, all of which impact the prevention and treatment of disease. Incorrect positioning and breathing techniques, failure to shake the inhaler, and premature closure of the device are the most common errors [9]. Furthermore, improper inhaler technique, while using inhaled corticosteroids, and inadequate throat rinsing and gargling after the use of inhaler usually raises the risks of adverse effects such as dysphonia and oral candidiasis. Therefore, the correct inhaler technique is important for managing these respiratory diseases [10].

Previous studies have reported that there is a high rate of incorrect inhalation techniques among patients with asthma and COPD. In one of the studies, 120 patients were enrolled, of whom 60 patients had asthma and COPD each, and 94.2% of patients made at least one mistake in using an inhaler. [11]. Another study by Aydemir et al. found that correct usage of DPIs was only 58.9% and MDIs was only 31.1% before training. However, after training, the rate of correct usage increased to 92.1% for DPIs and 45.2% for MDIs [12]. As many patients still use their inhalers incorrectly, a complete evaluation of inhaler techniques and training is necessary to confirm that patients are using their inhalers appropriately. This study aimed to assess the efficacy of training and counseling in improving inhaler technique and disease control in COPD and asthma patients.

## Materials and methods

A prospective, interventional study was conducted at the Department of Medicine, Pak Amirates Military Hospital, Rawalpindi, from October 2022 to March 2023. It was initiated after obtaining ethical approval from the Institutional Ethical Review Committee (IRB number: A/28/EC/436/2022) and informed consent from all participants. This study was designed to analyze the upsides of intervention on the status of the inhaler technique. A total of 150 participants were enrolled through a non-probability consecutive sampling technique.

This study enrolled patients with a confirmed diagnosis of COPD, as outlined by the Global Initiative for Chronic Obstructive Lung Disease criteria, or asthma, as defined by the Global Initiative for Asthma criteria. All patients were regular users of a prescribed inhaler device (MDI, MDI with spacer, and DPI) for treatment. The patients were between 18 and 65 years old, competent in understanding study procedures, and provided written informed consent for participation in the study. Patients who were experiencing acute exacerbations of COPD or asthma or had been hospitalized for these conditions were not included in the study. Individuals with comorbidities that could interfere with the intervention or assessment, cognitive impairments, and pregnancy were also excluded. Additionally, individuals who participated in other clinical trials, those with language problems limiting comprehension of the intervention, and those incapable of granting permission were excluded.

A total of 150 patients with COPD or asthma were incorporated into the study and divided into three groups based on the type of inhaler used (MDI, MDI + spacer, or DPI). Sociodemographic and clinical information such as age, gender, education status, smoking status, diagnosis, duration of disease, severity of disease, type of inhaler, and duration of inhaler use were gathered from patients. Patients were assured that all information and records would be kept confidential.

The inhaler technique was assessed using the checklist from the National Health Service (NHS) Liverpool Clinical Commissioning Group. According to this checklist (included in the Appendices as Table 4), each correct step was awarded 1 point, while incorrect or omitted steps received 0 points. There were seven steps in total, each scored as 1 or 0, resulting in a total possible score of 0-7. Based on the scores obtained, the inhaler technique was classified into the following three categories: a score of 0 to 3 indicated poor technique, a score of 4 to 5 suggested a moderate technique, and a score of 6 or 7 indicated good technique. To detect incorrect device application, each patient was asked to demonstrate the inhalation technique with prescribed devices to the medical professional by using a placebo device. As prescribed in the study protocol, correct use was assessed using predefined checklists for each inhaler type. Patients with incorrect techniques were provided with counseling and a physical demonstration of the proper method repeatedly and patients were asked to repeat these steps. They were also encouraged to ask questions for further clarification. The inhaler technique was re-evaluated using the same scoring system again after three months of training and counseling on inhalation techniques conducted by the same medical professional.

The asthma control test (ACT) was used to assess an individual’s control over their asthma. This self-administered questionnaire provides a numerical score that reflects the patient’s asthma management over the past four weeks. It consists of five questions that address different aspects of asthma control, such as the frequency of symptoms, nighttime awakenings, use of rescue medications, impact on daily activities, and self-assessment of control. Each question has a score ranging from 1 to 5, where 1 indicates poor control and 5 indicates good control. The total score ranges from 5 to 25, where a score of 20 or above indicates well-controlled asthma, a score of 16 to 19 suggests partially controlled asthma, and a score of ≤15 indicates poorly controlled asthma. The ACT score was recorded at the baseline visit and after three months of training and counseling on inhalation techniques. To check the severity of COPD, the COPD assessment test (CAT) was used. The assessment consists of eight questions that cover different aspects of COPD, such as cough frequency, phlegm production, chest tightness, breathlessness, limitations in physical activities, confidence in leaving home, sleep quality, and energy levels. Each question is scored from 0 to 5, with higher scores indicating a greater impact on the patient’s life. The total score ranges from 0 to 40, categorizing the impact of COPD as mild (0-10), moderate (11-20), severe (21-30), or very severe (31-40). The CAT score was recorded at the baseline visit and after three months of training and counseling on inhalation techniques.

Data were analyzed using SPSS version 26.0 (IBM Corp., Armonk, NY, USA). Descriptive statistics were presented as mean and standard deviation, while qualitative variables were presented as frequency and percentages. Qualitative baseline characteristics and outcomes were compared among different inhaler techniques using the chi-square test. Quantitative outcomes were compared using the analysis of variance test. Within the same inhaler technique pre- and post-counseling, quantitative outcomes were compared using paired t-test. For all the tests, a p-value <0.05 was considered statistically significant.

## Results

Of the 150 patients, 97 (64.7%) were males and 53 (35.3%) were females. There were 67 (44.7%) asthmatic and 83 (55.3%) COPD patients. The average age of the participants was 45.33 ± 12.62 years. Of these, 42 (43.3%) males had asthma and 55 (56.7%) males had COPD, while 25 (47.2%) females had asthma and 28 (52.8%) females had COPD. One-third of the study’s patients, 50 (33.3%), used each inhaler type: MDI, MDI with spacer, and DPI. Smoking status showed 33 (22%) current smokers, 72 (48%) non-smokers, and 45 (30%) ex-smokers. Most asthma cases were in the 20-35-year age group, 41 (61.1%), while the 51-65-year age group had the highest proportion of COPD patients, 48 (57.8%). Baseline demographic and clinical details of patients using different inhalers are reported in Table 1 and Table 2.

**Table 1:** Distribution of demographic and clinical characteristics of patients by inhaler types. MDI = metered-dose inhaler; DPI = dry powder inhaler; BMI = body mass index; COPD = chronic obstructive pulmonary disease

Characteristics	MDI	MDI + spacer	DPI	P-value
Age (years)	44.20 ± 12.47	45.48 ± 12.96	46.32 ± 12.58	0.702
BMI (kg/m^2^)	27.09 ± 4.26	27.22 ± 3.47	25.78 ± 3.24	0.097
Duration of disease (years)	5.97 ± 1.75	6.15 ± 2.18	5.56 ± 2.14	0.329
Duration of therapy with inhaler (years)	3.09 ± 1.02	2.96 ± 1.02	2.82 ± 1.12	0.453
Age groups				0.774
20–35 years	13 (26.0%)	13 (26.0%)	15 (30%)	
36–50 years	18 (36.0%)	16 (32.0%)	12 (24.0%)	
51–65 years	19 (38.0%)	21 (42.0%)	23 (46.0%)	
Gender				0.890
Male	33 (66.0%)	33 (66.0%)	31 (62.0%)	
Female	17 (34.0 %)	17 (34.0%)	19 (38.0%)	
Smoking status				0.471
Current smoker	15 (30.0%)	08 (16.0%)	10 (20.0%)	
Ex-smoker	12 (24.0%)	16 (32.0%)	17 (34.0%)	
Non-smoker	23 (46.0%)	26 (52.0%)	23 (46.0%)	
Education status				0.293
Illiterate	13 (26.0%)	17 (34.0%)	11 (22.0%)	
Primary	18 (36.0%)	14 (28.0%)	11 (22.0%)	
Secondary	12 (24.0%)	11 (22.0%)	21 (42.0%)	
Graduation	07 (14.0%)	08 (16.0%)	07 (14.0%)	
Disease				0.599
Asthma	20 (40.0%)	25 (50.0%)	22 (44.0%)	
COPD	30 (60.0%)	25 (50.0%)	28 (56.0%)	

**Table 2:** Comparison of correct inhaler technique steps done before and after counseling by patients using different inhalers. MDI = metered-dose inhaler; DPI = dry powder inhaler

	MDI		MDI + spacer		DPI	
Checklist steps	Pre-counseling n (%)	Post- counseling n (%)	Pre-counseling n (%)	Post- counseling n (%)	Pre-counseling n (%)	Post- Counseling n (%)
Step 1	41 (92.0%)	49 (98.0%)	38 (76.0%)	48 (96.0%)	48 (96.0%)	50 (100%)
Step 2	39 (78.0%)	48 (96.0%)	46 (92.0%)	50 (100%)	42 (84.0%)	47 (94.0%)
Step 3	26 (52.0%)	46 (92.0%)	22 (44.0%)	44 (88.0%)	38 (76.0%)	45 (90.0%)
Step 4	41 (92.0%)	48 (96.0%)	36 (72.0%)	48 (96.0%)	34 (68.0%)	46 (92.0%)
Step 5	21 (42.0%)	43 (86.0%)	26 (52.0%)	41 (82.0%)	27 (54.0%)	41 (82.0%)
Step 6	19 (38.0%)	38 (76.0%)	31 (62.0%)	45 (90.0%)	21 (42.0%)	41 (82.0%)
Step 7	32 (64.0%)	42 (84.0%)	29 (58.0%)	43 (86.0%)	36 (72.0%)	48 (96.0%)

**Figure 1:**
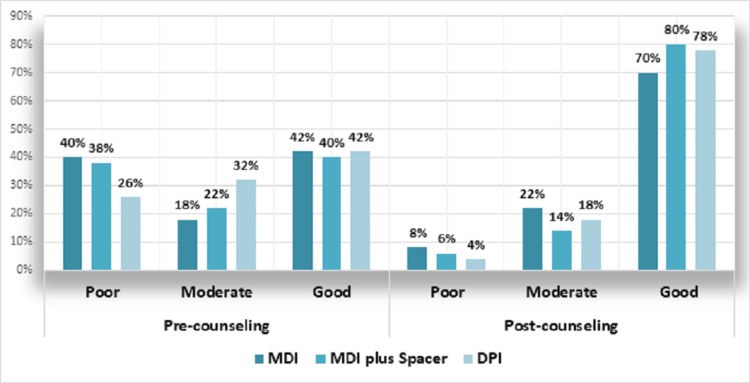
Comparison of inhaler technique response before and after counseling across different inhaler modalities. MDI = metered-dose inhaler; DPI = dry powder inhaler

At baseline, patients using MDI exhibited the highest rate of poor technique at 20 (40%), whereas those using DPI had the highest rate of good technique, with no significant difference in technique across inhaler types (p = 0.426). Post-counseling, the minimum poor technique was observed in DPI users at 02 (4%), and the maximum poor technique was in MDI users at 04 (8%). Conversely, the maximum good technique was noted in MDI with spacer users at 40 (80%), while the minimum good technique was reported by MDI users at 35 (70%) (p = 0.750) (Figure 1).

Although no significant differences were noted in inhaler technique or disease control scores across MDI, MDI with spacer, and DPI groups before and after counseling (p > 0.05), post-counseling improvements were substantial: MDI technique scores improved from 4.4 to 6.1, MDI with spacer from 4.56 to 6.26, and DPI from 4.92 to 6.24 (p < 0.001 for each). CAT scores decreased significantly for MDI from 23.4 to 20.5, MDI with spacer from 23.92 to 20.96, and DPI from 24.89 to 21.96, while ACT scores increased for MDI from 16.4 to 18.0 (p = 0.002), MDI with spacer from 17.29 to 19.04, and DPI from 16.42 to 18.37 (p < 0.001 for both), indicating a significant improvement in managing both COPD and asthma post-intervention (Table 3).

**Table 3:** Comparison of inhaler technique steps score and disease control scores before and after counseling across different inhaler modalities. MDI = metered-dose inhaler; DPI = dry powder inhaler; CAT = COPD assessment test; ACT = asthma control test

	MDI		MDI + spacer		DPI	
	Pre-counseling	Post-counseling	Pre-counseling	Post-counseling	Pre-counseling	Post-counseling
Technique total step score	4.38 ± 2.31	6.06 ± 1.46	4.56 ± 2.20	6.26 ± 1.13	4.92 ± 1.96	6.24 ± 1.09
ACT score	16.37 ± 4.01	17.98 ± 3.22	17.29 ± 3.43	19.04 ± 3.11	16.42 ± 2.83	18.51 ± 2.54
CAT score	23.43 ± 7.22	20.47 ± 7.27	23.92 ± 8.54	20.96 ± 8.59	24.89 ± 6.44	21.96 ± 6.54

## Discussion

In the realm of respiratory medicine, the optimization of inhaler techniques remains an important element in the management of respiratory diseases. Recognizing the inhaler as a pivotal instrument for respiratory therapy, its efficacy is inherently linked to the precision of the inhalation technique, the inhaler type, and patient adherence. Proper application of these devices plays a significant role in terms of disease treatment efficacy but also in elevating the patient’s QoL. In this study, a structured intervention on inhaler usage was imparted through hands-on demonstrations, avoiding printed aids in favor of direct, physical instruction. The physician played a pivotal role in this educational intervention, utilizing a standard checklist provided by the NHS to guide and correct inhaler techniques [13].

The effectiveness of counseling in enhancing inhaler technique is affirmed by the notable improvements observed in this study, particularly in the execution of specific steps with the MDI. Before counseling, most patients adeptly executed Step 1 (removing the cap on the mouthpiece) and Step 4 (securing the mouthpiece with a good seal), with an accuracy rate of 92%. Conversely, Steps 2 and 7 (holding the inhaler upright and shaking well, and breathing out gently post-inhalation) showed lower adherence rates at 78% and 64%, respectively. Following the counseling, these steps saw improvement rates jump to 96% and 84%. Comparatively, the study by Thakkar et al. reported high pre-counseling adherence to these crucial steps, with subsequent post-counseling enhancements demonstrating the consistent efficacy of educational interventions [14]. Yet, it was Steps 3, 5, and 6 (breathing out gently, initiating slow inhalation while pressing the canister, and holding the breath post-inhalation) where our study observed the most dramatic improvements, from 52%, 42%, and 38% pre-counseling to 92%, 86%, and 76% post-counseling, respectively. Thakkar et al. corroborated these findings, particularly noting significant post-counseling advancements in the correct execution of Steps 3 and 6. Şen et al. (2006) revealed that pre-intervention, correct MDI usage was observed in 36.17% of cases, whereas post-intervention, proper technique usage increased to 65.5% [15].

In this analysis, we discern a pattern where patients using the MDI with spacer inhalers initially displayed challenges in executing several steps correctly. Specifically, steps that require fine motor skills and coordination, such as Step 3 (breathing out gently), Step 5 (holding the spacer level and pressing down firmly on the canister), Step 6 (removing the spacer from the mouth and breathing out gently), and Step 7, (removing the inhaler from the spacer) were less frequently performed correctly compared to the simpler action of Step 2 (inserting the inhaler into the spacer). These observations suggest the MDI with spacer’s operational complexity might pose difficulties for patients, highlighting the critical need for thorough training. Post-intervention, we observed significant improvements; the correct performance of Step 3 nearly doubled to 88%, and similar enhancements were seen with the execution of Steps 5, 6, and 7. These improvements indicate that with proper counseling and demonstration, patients can overcome the complexities of using MDI with spacer inhalers. These findings echo the outcomes of other studies, which also noted particular difficulties patients faced with Step 3 (preparing the device for use) and Step 7 (concluding the inhalation process). Patients frequently exhibited challenges with particular inhalation technique steps such as maintaining breath-hold for 10 seconds following medication inhalation, as well as performing a gentle exhalation before inhalation. Optimal exhalation to the point of comfort before inhalation is crucial; it minimizes airway air content, thereby augmenting the available space for inhalation, which is essential for the effective delivery of the therapeutic agent to its intended target within the respiratory tract. Therefore, clinical instruction should underscore the importance of these steps when educating patients on the utilization of inhalation devices [16,17]. Similar errors were observed in this study among individuals employing MDI and MDI with a spacer.

For patients utilizing DPIs, the study revealed a notable difficulty in performing Step 4 (pressing the side buttons to pierce the capsule), Step 5 (breathing out and then inhaling through the device), and Step 6 (holding breath post-inhalation), with baseline correctness being 68%, 54%, and 42%, respectively. The counseling intervention markedly enhanced the execution of these steps, with each seeing an increase in correctness to over 80%. This remarkable improvement suggests that patients were able to grasp and apply the more complex aspects of DPI use with effective counseling. In comparison, the study by Thakkar et al. highlighted similar challenges, particularly with Step 6, which showed the most significant improvement from 28% to 78% post-intervention, underscoring the critical role of patient education in mastering the DPI technique [14].

The study delineated marked progress in inhaler technique across device types after intervention. Initially, 40% of MDI users exhibited poor technique, with only 42% demonstrating good technique. This improved significantly post-intervention, with 70% attaining good technique. Among DPI users, initial poor technique was noted in 26%, and 42% had good technique, which increased to 78% post-intervention. Users of MDI with a spacer displayed the most notable enhancement, with good technique observed in 80% post-counseling, up from the least pre-counseling. Thakkar et al. also observed a significant increase, particularly with MDI users, where post-intervention good technique soared to 91.49% [14].

This study highlighted significant improvements in inhaler technique and ACT scores among young adults (20-35 years), with notable increases observed (p = 0.002 and p = 0.001, respectively). This aligns with findings from other studies such as those by Shrestha et al. (2013), Arora et al. (2014), and Thakkar et al. (2021), which have shown that elderly patients tend to exhibit more errors in inhaler technique compared to younger and middle-aged patients. The challenges faced by older individuals often stem from forgetfulness of the proper steps or the presence of comorbidities that interfere with effective inhaler use [14,18,19].

Education also played a crucial role, with individuals having only primary education showing significant improvements in technique (p < 0.001), challenging the notion that higher education is a prerequisite for mastering inhaler use. This is particularly significant as previous studies often highlight better outcomes in educated individuals who are presumably better able to understand instructional materials. However, our findings suggest that effective counseling, tailored to meet the educational levels of patients, can bridge this gap.

Many previous studies have indicated that individuals with higher educational levels tend to grasp inhaler techniques more effectively than those who are illiterate due to their ability to understand and follow instructions provided in the leaflets included with inhaler packaging [11,14,19]. However, this study found that patients with only primary education exhibited significant improvements in their inhaler technique following counseling (p < 0.001). This finding challenges the idea that higher education is necessary for effective inhaler use. It suggests that hands-on sessions and counseling, customized to accommodate various educational backgrounds, are crucial in reducing disparities in inhaler technique.

This study highlighted notable improvements in inhaler technique post-counseling along with significant improvements in managing symptoms of COPD and asthma. CAT scores showed significant reductions across all inhaler types post-intervention, with MDI users’ scores decreasing from 23.4 to 20.5, MDI with spacer from 23.92 to 20.96, and DPI from 24.89 to 21.96. For asthma management, ACT scores increased substantially, indicating improved control; scores for MDI, MDI with spacer, and DPI users improved to 18.0, 19.04, and 18.37, respectively (p < 0.001 for both). These results demonstrate that effective counseling enhances not only the technique but also the overall health outcomes for respiratory disease patients.

This study had several limitations including a single-center design with a small sample size, potentially affecting the generalizability of the findings. Moreover, the counseling was delivered only once at baseline, possibly not capturing long-term adherence or the full effect of the training. However, the study had notable strengths, such as an excellent follow-up rate which ensured a reliable dataset. The inhaler technique steps were demonstrated by a trained physician and repeated multiple times, with patients asked to perform these steps two to three times, enhancing the learning process. The study not only observed improvements in inhalation techniques but also evaluated how these teaching sessions impacted disease symptom severity. Future research should involve a larger, more diverse population across multiple centers, especially in regions with varied socioeconomic statuses to intensify the understanding of educational interventions on inhaler techniques, which is vital for regions where respiratory conditions are widespread and health education is essential.

## Conclusions

The study’s interventions led to notable improvements in inhaler technique across MDI, MDI with spacer, and DPI users, with significant enhancements in key inhalation steps post-counseling. Despite initial demographic variances, the interventions were effective across all subgroups, indicating that tailored counseling is crucial in overcoming educational barriers to proper inhaler use. The marked increase in correct technique application and the resulting better disease control signifies the success of the interventions. These findings highlight the importance of structured counseling and reinforce the need for such educational programs in clinical settings to improve disease management in respiratory patients. Future research should focus on the longitudinal impact of counseling and the expansion to multicenter studies for broader demographic insights.

## Appendices

**Table 4:** Standard checklist of steps of different types of inhalers. [14].

Metered-dose inhaler (MDI) checklist	
Step 1	Detach the cap from the mouthpiece
Step 2	Keep the inhaler vertical and shake thoroughly
Step 3	Exhale slowly to a comfortable level
Step 4	Position the mouthpiece in your mouth and seal the lips tightly around it
Step 5	Inhale slowly and simultaneously press down on the canister firmly
Step 6	Hold your breath for up to 10 seconds, and then remove the inhaler from your mouth
Step 7	Exhale softly, reattach the cap, and if another dose is needed, wait 1 minute before repeating steps 3 to 6
Metered-dose inhaler (MDI) + spacer checklist	
Step 1	Remove the cap from the inhaler, hold it vertically, and shake vigorously
Step 2	Insert the inhaler upright into the spacer’s designated opening
Step 3	Exhale comfortably to a relaxed extent
Step 4	Place the spacer’s mouthpiece between your teeth without biting and seal your lips tightly around it
Step 5	Keep the spacer horizontal, activate the inhaler to release a puff, inhale slowly and deeply, hold your breath for 10 seconds, then breathe normally for four breaths
Step 6	Detach the spacer from your mouth, exhale gently, and if another dose is needed, repeat steps 3 to 5 after shaking the inhaler again and waiting for one minute
Step 7	Remove the inhaler from the spacer, replace the cap, and ensure the patient is aware of how to clean the spacer monthly if applicable
Dry powder inhaler (DPI) checklist	
Step 1	Remove the cap and tilt the mouthpiece backward to open the device
Step 2	Extract a capsule from the blister pack and place it at the base of the device
Step 3	Close the device by pushing the mouthpiece forward until it clicks over the capsule chamber
Step 4	Press the side buttons inward to pierce the capsule, then release the buttons
Step 5	Exhale fully, position your lips around the mouthpiece, and inhale deeply—you will notice a whirring sound during inhalation
Step 6	Hold your breath for 5–10 seconds, exhale, and check if the capsule is empty. If not, remove the spent capsule, close the device if the powder remains, and inhale the rest
Step 7	Rinse your mouth with water after use to clean out any residual powder
